# Influence of ocean circulation and the Kuroshio large meander on the 2018 Japanese eel recruitment season

**DOI:** 10.1371/journal.pone.0223262

**Published:** 2019-09-27

**Authors:** Yu-Lin K. Chang, Yasumasa Miyazawa, Michael J. Miller, Katsumi Tsukamoto

**Affiliations:** 1 Application Laboratory, Japan Agency for Marine-Earth Science and Technology, Yokohama, Japan; 2 Department of Aquatic Bioscience, The University of Tokyo, Tokyo, Japan; Universidade de Aveiro, PORTUGAL

## Abstract

Japanese eel (*Anguilla japonica*) recruitment to Japan was very low during the early 2017−2018 recruitment season when most glass eels are usually caught, but catches increased in the late recruitment season when recruitment usually decreases. Concurrently, the Kuroshio meander south of Japan had formed again after the previous event ended in 2005. The role of the large meander and ocean circulation features such as the North Equatorial Current (NEC) in the unusual 2017−2018 Japanese eel recruitment timing-pattern was investigated using a three-dimensional particle tracking model that simulated swimming behaviors of virtual larvae (v-larvae) in addition to their drift in ocean currents. Four recruitment seasons were selected for when the Kuroshio large meander was present (2004−2005, 2017−2018) or absent (2009−2010, 2015−2016), and when NEC was shifted north (2004−2005, 2015−2016) or south (2009−2010, 2017−2018). The simulated recruitment timing-patterns were similar to the actual recent-year recruitment, with no early recruitment period v-larvae arrival to southern Japan and increased late period recruitment occurring. Rather than being related to the presence of the Kuroshio large meander, the late arrival appeared to be caused by a southward shifted, weak North Equatorial Current near the spawning area, a longer Subtropical Countercurrent eddy region retention time, and a weak Kuroshio during the early migration and recruitment period of those years. In the late recruitment period, the Kuroshio was stronger than other selected years near the East China Sea and south of Japan and v-larvae were transported more rapidly. The Kuroshio large meander may influence local eel recruitment in Japan, and the recirculation formed by the large meander could potentially enhance recruitment to the Tokai region. Oriented (northwestward) swimming v-larvae were less affected by the large meander, and showed higher recruitment success than those using along-current swimming. Although the Kuroshio large meander did not seem to be responsible for the unusual recruitment pattern in 2018, how Japanese eel larvae and glass eels actually cross out of the Kuroshio and reach coastal waters in Japan remains to be explored.

## Introduction

Anguillid eels are widely distributed in the Indo-Pacific and North Atlantic regions where the juveniles live in freshwater and estuarine habitats [1, 2]. Their adults migrate offshore to spawn over deep water and their larvae, called leptocephali, have a long larval dispersal stage that varies among tropical and temperate zones, but all species metamorphose into recruitment stage glass eels before they migrate into inshore waters [3–5]. World eel recruitment has significantly declined in the past few decades [6], raising conservation concerns about their populations and what has caused the declines. Overfishing and habitat loss due to human activities have been considered to be one of the primary factors causing the eel recruitment decline [7]. Changes in ocean circulation or oceanic conditions may also significantly affect eel recruitment according to various types of information about the Atlantic eels [8–11] and Japanese eels [12–16], or both types of factors have likely contributed [17].

**Fig 1:**
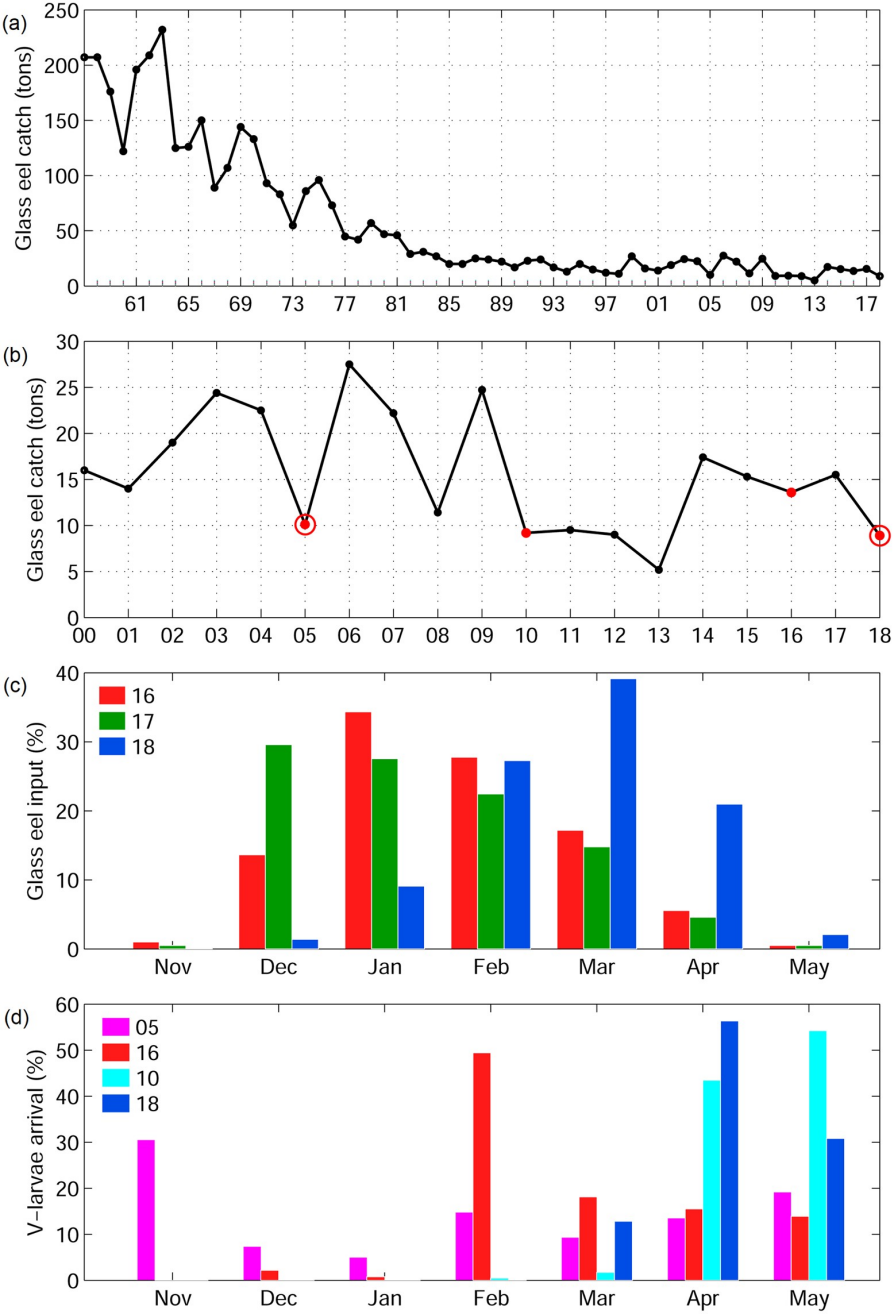
Observed glass eel catches from 1958 to 2018 in Japan (a), since 2000, with the years used in the simulations shown in red and Kuroshio large meander years with red circles (b), and monthly glass eel catches/input (showing percentage of annual catch) in Japan from 2016 to 2018 (c). Simulated monthly v-larvae arrival (%) to southern Japan (d).

The Japanese eel, *Anguilla japonica*, is one of the most important eel species for fisheries and aquaculture, and it has been listed as endangered on the IUCN red list [18]. Among the Northern Hemisphere species of eels that have all experienced declines, the Japanese eel seems to have shown the earliest recruitment declines that started in the 1970s [19], and recruitment to Japan was declining since the 1960s (Fig 1a). After about 2010, the annual recruitment had decreased by as much as 90% compared to eel catches in the 1960s [18]. The growth habitats of the Japanese eel are distributed across East Asia, and their spawning area is located along the West Mariana Ridge within the westward flowing North Equatorial Current (NEC) [20, 21] in the western North Pacific (WNP). The NEC forms a zone of westward flow from about 10° to 20°N, which then bifurcates into northward Kuroshio and southward Mindanao Current flows [22, 23]. The eel larvae are carried primarily by the NEC and Kuroshio toward their growth habitats in East Asian rivers and estuaries, although it is possible that some active swimming occurs [5], which might increase recruitment success [24].

The glass eel recruitment season of the Japanese eel in East Asia is from November to May of the next year. The glass eel catches in Japan generally reach their maximum levels early in the recruitment season in December or January and decrease after that (Fig 1c). In December 2017, the glass eel catch was less than 5% of the previous year, and these extremely low catches also occurred in Taiwan (https://goo.gl/JmU54s). Due to the lack of eel catches in the early recruitment season, the market transaction price of glass eels in Japan had reached a historical maximum of 30,000 USD per kilogram (the average price was about 10,000 USD per kilogram from 2003 to 2017). The drastic decline of eel catches was a big news story that was covered by the media worldwide. During the same period, the Kuroshio large meander was occurring. The Kuroshio large meander forms when the path of the current flow turns sharply to the south and then turns back north in the region south of Japan [25]. The Kuroshio large meander does not occur regularly and the previous event was in 2004 when it lasted for one year. The meander formed again in the summer of 2017 and was still present into 2019.

**Fig 2:**
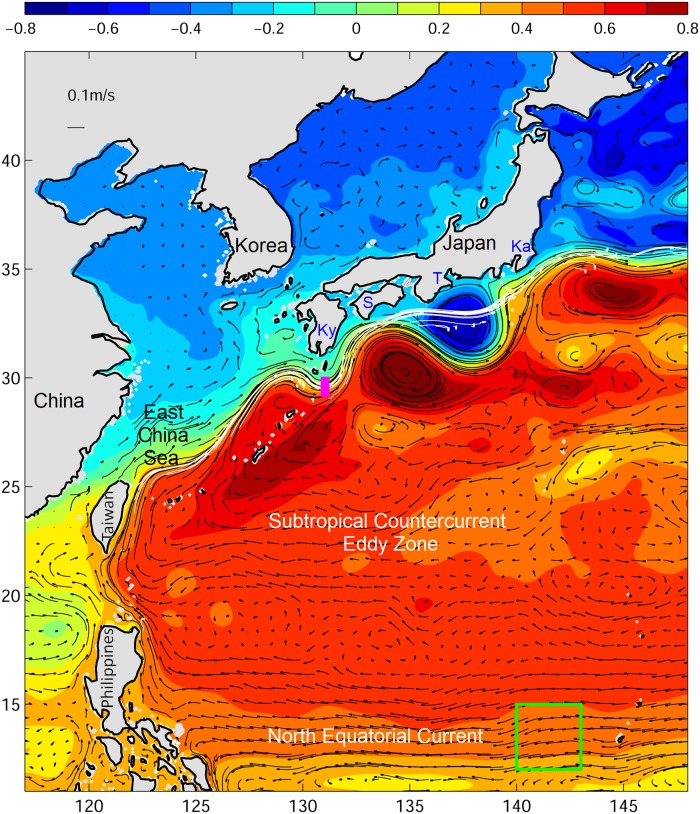
Mean ocean circulation during the 2018 eel catch season (November 2017 to May 2018). Color shading is sea surface height (m), and vectors are 50−250 m mean ocean currents. Ky, S, T, and Ka show the Kyushu, Shikoku, Tokai, and Kanto regions of Japan, respectively. The green box shows the spawning area where v-larvae were released. Magenta box south of Kyushu shows the release region for examining the effect of the Kuroshio large meander with different swimming behaviors. The white line shows where the ocean current was stronger than 0.4 m/s during the 2010 Kuroshio non-large meander eel catch season.

The Kuroshio meander path does not change the current path along the entire coast of Japan, but mainly influences the path near the Tokai region (Fig 2). The Kuroshio meander can extend about 300−500 km from the shore (Fig 2). The region inside the meander from the Kuroshio to the coast forms a cyclonic circulation cell that contains cooler water than in the Kuroshio [26]. Several possible factors that might cause the large meander to occur have been proposed [27–29], but the exact cause has remained unknown. Due to the drastically different offshore position of the Kuroshio, there has been speculation in the media that the meander could potentially influence glass eel transport and entry into coastal waters, and some extreme low recruitment events were sometimes attributed to the Kuroshio large meander (https://goo.gl/QTEp45).

However, several other oceanic factors have been investigated as possibly affecting interannual levels of recruitment success using larval transport modelling simulations, which are not related to the large meander. These included El Niño or La Niña conditions [15, 30], NEC bifurcation latitude [12, 14], offshore mesoscale eddies [31, 32]. Various effects were found, with especially the NEC and Kuroshio seeming to have some influence on recruitment success. While it is not known if the Japanese eel larvae use active swimming, it seems to be required for the larvae to swim to be able to cross out of the Kuroshio and reach recruitment areas, and transport modelling simulations suggest it may be able to increase recruitment success if it is used during various stages of the larval migration [24].

While none of the factors investigated in modelling studies have been generally accepted as controlling interannual recruitment variations, the 2017−2018 recruitment season was unusual for its timing of glass eels catches. In contrast to the early recruitment season, eel catches started to increase and reached a maximum in March 2018 during the late recruitment season when eel catches usually decrease. The monthly catch in March and April 2018 were 2−3 times higher than in those months of the previous two years (Fig 1c). The annual recruitment in 2018 remained low due to the extremely low catch in the early recruitment season. However, it was probably not the lowest annual catch due to the significant increase in recruitment late in the season (Fig 1c).

The present study investigates the unusual recruitment timing that was observed in 2018 to evaluate if the Kuroshio large meander might have been a causative factor or if other previously identified ocean circulation factors were possible influences. The extremely low catch in the early recruitment season and the exceptionally high catch in the late recruitment period are examined in relation to the presence or absence of the Kuroshio large meander and different ocean current conditions in the WNP using larval transport modelling. The model reanalysis and observed glass eel catches were used to evaluate the effect of ocean current characteristics on the late glass eel arrival by comparing two recruitment years when the Kuroshio large meander was present and two years when it was absent. Because the bifurcation latitude of the NEC has been suggested to influence eel larvae recruitment to East Asia [12, 14], the two non-meander years were chosen to be either NEC north-shifted or south-shifted years for comparison to the meander years. The dispersal of virtual larvae (v-larvae) in the WNP in 2017−2018 and three comparison recruitment seasons/years with various oceanic conditions were simulated based on a three-dimensional (3D) particle tracking method, in which swimming behavior was included in addition to transport by ocean currents. Separate simulations were conducted to examine the effect of directional swimming in relation to the Kuroshio large meander, because this type of swimming was found to be effective for crossing out of the Kuroshio in a previous study [24]. Biological factors affecting mortality (i.e., feeding success or predation) were not considered in the present work that focused on the effects of ocean circulation.

## Data and methods

### Observations

Annual glass eel recruitment data from 1957 to 2018 were obtained from the Japan Fisheries Agency, which included all reported glass eel catches in Japan each year. The official definition used by the Japan Fisheries Agency is that the recruitment season is from November to May of the next year. Monthly data is available from the Japan Fisheries Agency since 2016 that includes glass eel catch/input data from each prefecture, which can also include some imported glass eels from China and Taiwan. We used these monthly data pooled for all of Japan as a rough approximation of the levels and timing of recruitment to Japan/East Asia, because the abundance of catches in each area are likely geographically interrelated, and at least the low catches/inputs in Japan are directly indicative of low recruitment levels there. The Kuroshio speed anomaly was estimated from tide gauge sea level data based on geostrophy [33], and tide gauge data from the Oodori and Naze stations south of Japan were obtained from the Japan Coast Guard. The data is also available from a Kuroshio monitoring site provided by the University of Tokyo (https://ovd.aori.u-tokyo.ac.jp/tides/).

### Ocean reanalysis data: JCOPE2

The data-assimilative ocean circulation model known as the Japan Coastal Ocean Predictability Experiment 2 (JCOPE2) provided the three-dimensional currents and hydrological fields that were used for particle tracking in the present study. JCOPE2 was constructed from the Princeton Ocean Model with a generalized coordinate system [34]. The model domain of JCOPE2 encompasses most of the WNP (10.5–62°N and 108–180°E), with a horizontal resolution of 1/12° (8–9 km) and 46 vertical layers. The lateral boundary conditions are determined from the basin-wide model, using a one-way nesting method. The external forcing to drive JCOPE2 includes wind stresses and net heat/freshwater fluxes at the sea surface that are converted from the six-hourly atmospheric reanalysis produced by the National Centers for Environmental Prediction/National Center for Atmospheric Research. Satellite sea surface temperature, sea surface height, and in situ temperature and salinity data were assimilated into the model based on a three-dimensional variational method [34]. The daily JCOPE2 reanalysis fields cover the period from January 1993 to the present. Comparison of simulated trajectories of passive particles carried by JCOPE2 and observed trajectories was performed in a previous study [12], which showed a satisfactory performance of JCOPE2 in simulating the three-dimensional circulation across the WNP

### Particle-tracking scheme

A particle-tracking method was used to simulate the movement of v-larvae of the Japanese eel during 4 recruitment seasons/years. The particles were carried by ocean currents in addition to having their own swimming behavior within the ocean currents simulated by the JCOPE2 reanalysis. The specific v-larvae swimming behavior was introduced into the experimental setup that was based on the particle-tracking scheme developed by Ohashi and Sheng [35]. The tracking scheme was based on the fourth-order Runge–Kutta method [36] with a tracking time step of three hours, which was chosen corresponding to model resolution and ocean current speed. A shorter time step would be required for a higher model resolution. The same tracking scheme was used in previous studies for investigating the migration of Japanese eel larvae and adults in the WNP and was also used in simulations of the long-distance migration of adult American eels in the Atlantic Ocean [37, 38].

A random walk displacement was included in all experiments to represent unresolved sub-grid turbulent flow and other local processes [35]. The estimated maximum horizontal and vertical displacements due to the random walk were 600 m and 20 m during the simulation period, respectively. The duration of day and night were determined by the time of sunrise and sunset, which were set seasonally. Day length in spring and autumn was set to 12 hours (6 am to 6 pm) and was shortened to 10 hours (7 am to 5 pm) in winter (December–February), and lengthened to 14 hours (5 am to 7 pm) in summer (June–August).

### Experimental design

Numerical experiments were conducted to examine the late arrival of v-larvae transport to East Asia during the 2017−2018 recruitment season in comparison to 3 other recruitment seasons (see below). The release region and time of v-larvae were chosen according to the observed spawning area and season based on the collections of Japanese eel eggs, newly hatched preleptocephali, and spawning-condition adults along of the southern West Mariana Ridge [21, 39]. The particles (v-larvae) were released at locations spread over the region of 140 to 143°E and 12 to 15°N (one v-larvae at each location) with a separation distance of 10 km in both zonal and meridional directions. Leptocephali estimated to be 10–40 days old were found in May, June and July during sampling surveys [39, 40], and the eggs and were caught a few days prior to the new moon [21, 39]. The release times were set from May to July at 2−4 days prior to the new moon during the three-month period. About 8,000 v-larvae were released each year. The tracking duration was set to cover the eel recruitment season in East Asia from November to the following May. Following previous studies [12, 13], horizontal swimming in the same direction as the ambient current and DVM were set to be age-dependent and included a linear increase of body length. Because the 24-hour continuous swimming assumed in previous studies [12, 31, 32] would be energy consuming during long-term migration, and the newborn larvae may just drift with ocean currents while they are feeding and growing, v-larvae were set to start swimming when they reached 30-days old, and they only swam during daytime and passively drifted in ocean currents at night [24]. Swimming speed was set to increase 0.075 cm/s per day from zero at starting at 30 days after release, with a maximum speed of 15 cm/s that would be reached when the larvae reach their maximum size (60 mm TL) [41]. DVM started on day 0 and became linearly deeper from 50 m to 250 m during the day with the age of v-larvae, and stayed at 50 m at night. If the larvae moved over bottom depths that are shallower than the diving depth, they were set to stay at 10% of water depth above the seafloor. The swimming direction of the v-larvae was the same as the local flow when water depths were greater than 100 m as in the previous studies [12, 13]. When the v-larvae entered coastal and shelf waters with water depths shallower than 100 m, they were set to search for lower salinity and swim toward low-salinity coastal waters [42], although in nature the larvae in nearshore waters would actually have already transformed into glass eels. Directional swimming to the northwest was found to increase the recruitment success of v-larvae of the Japanese eel [24], but it is not known if the larvae actually use this behavior in the ocean and this would bias the experiment, so this type of directional swimming was not used in the main part of the present study examining early and late season larval transport. The passive drift could also be used to examine the effect of ocean currents, but a previous study showed that passive transport of Japanese eel larvae was too slow compared to the estimated migration duration from observations [12], so passive drift was not applied in present study of the timing of arrival in relation to the Kuroshio large meander.

The selection of years for the simulations to examine the role of the Kuroshio large meander was based on the state of the Kuroshio meander (present or absent, Table 1). The bifurcation of the NEC was also considered because of its influence on eel larvae migration [12–14]. As a result, the years, 2004−2005, 2009−2010, 2015−2016, and 2017−2018 were chosen. The years 2004−2005 and 2017−2018 were the only two large meander events in the past 20 years, and the NEC bifurcation was shifted southward in 2018, whereas it was at a northern latitude in 2004. The non-large meander Kuroshio is the typical condition, and the year 2009 was chosen because the NEC was recorded at its southernmost location (8.3°N). The year 2016 was selected because the NEC was shifted to the north and there was monthly eel catch data available (monthly data was available since 2016). The simulation period was split into three periods for analysis, which were the early migration period from May to October, the early recruitment season from November to the coming January when glass eel catches were generally increasing, but were extremely low in 2018, and the late recruitment season from February to April when glass eel catches usually decrease, yet they peaked during that period in 2018. For convenience, each of the 4 simulation periods will usually be referred to in the text and figure labels by the second year of each recruitment season (i.e. 2005, 2010, 2016, 2018).

**Table 1:** List of experiments and the corresponding oceanic conditions used in each simulation for the main experiments on the effect of the presence of the Kuroshio large meander and swimming sensitivity experiments. For the meander experiments, release dates were 2–4 days before the new moon of each month during the spawning period in the selected years, and for the sensitivity cases, v-larvae were released in the Kuroshio south of Japan during the peak recruitment season. The numbers listed in the parenthesis of the North Equatorial Current (NEC) region are the mean NEC bifurcation latitudes. Both Kuroshio large meander (LM) and no large meander years (NLM) were used in the simulations.

Year	2005	2018	2010	2016
Kuroshio	LM	LM	NLM	NLM
Default	V-larvae released at spawning area (140−143°E, 12−15°N)			
NEC*	North (12.2)	South (9.9)	South (10.0)^+^	North (12.2)
Release dates	15−17 May	22−24 May	20−22 May	14−16 May
	14−16 June	20−22 June	19−21 June	12−14 June
	13−15 July	17−19 July	18−20 July	12−14 July
Sensitivity	V-larvae released at south of Japan (130.9−131.1°E, 29.2−30°N)			
Release dates	5, 15, 25 January			
	5, 15, 25 February			
	5, 15, 25 March			

*NEC bifurcation latitude was averaged from May to October

^+^NEC shifted to its recorded southernmost location of 8.4°N during part of the period

In order to examine the effect of the Kuroshio large meander on eel recruitment to Japan, and understand if larvae need to swim in an oriented direction to cross out of the Kuroshio, an additional set of sensitivity experiments was conducted in which v-larvae were released south of Japan (magenta box in Fig 1), with the larvae using various swimming behaviors. Swimming seems to be a requirement to cross out of strong western boundary currents such as the Gulf Stream, East Australian Current, and Kuroshio [5] and it appears effective for Japanese eel larvae [24]. The same years were chosen for the simulations and they were pooled according to the meander condition (large meander years in 2005 and 2018 and non-large meander years in 2010 and 2016). The release times were set on 5, 15, and 25 of January, February and March during the peak eel catch period in Japan, and the initial age of v-larvae was set to be 240 days. The DVM behavior of the larvae followed the settings described above. To evaluate different types of directional swimming or speeds, the sensitivity experiments included northwestward swimming, 24-hour northwestward swimming, and half-speed northwestward swimming along with the default along-current swimming. Successful recruitment was defined when v-larvae entered the continent shelf (bottom depths <200 m). For areas with a sharp slope next the coast with no continental shelf, v-larvae that arrived at 3 grid points (or closer) from the coast were counted as successful recruitment. Eddy occurrence in the Subtropical Counter Current (STCC) region was identified using the Okubo-Weiss Method [43, 44]. Percentage of eddy presence was calculated by the ratio of eddy areas within the defined STCC region. The average time taken from the spawning area to each particular region was calculated by taking the mean age of v–larvae that appeared at each individual grid location. Visitation Frequency was the number of particles that reach a particular grid location, normalized by the total number of particles.

## Results

### V-larvae distribution in early and late recruitment periods

**Fig 3:**
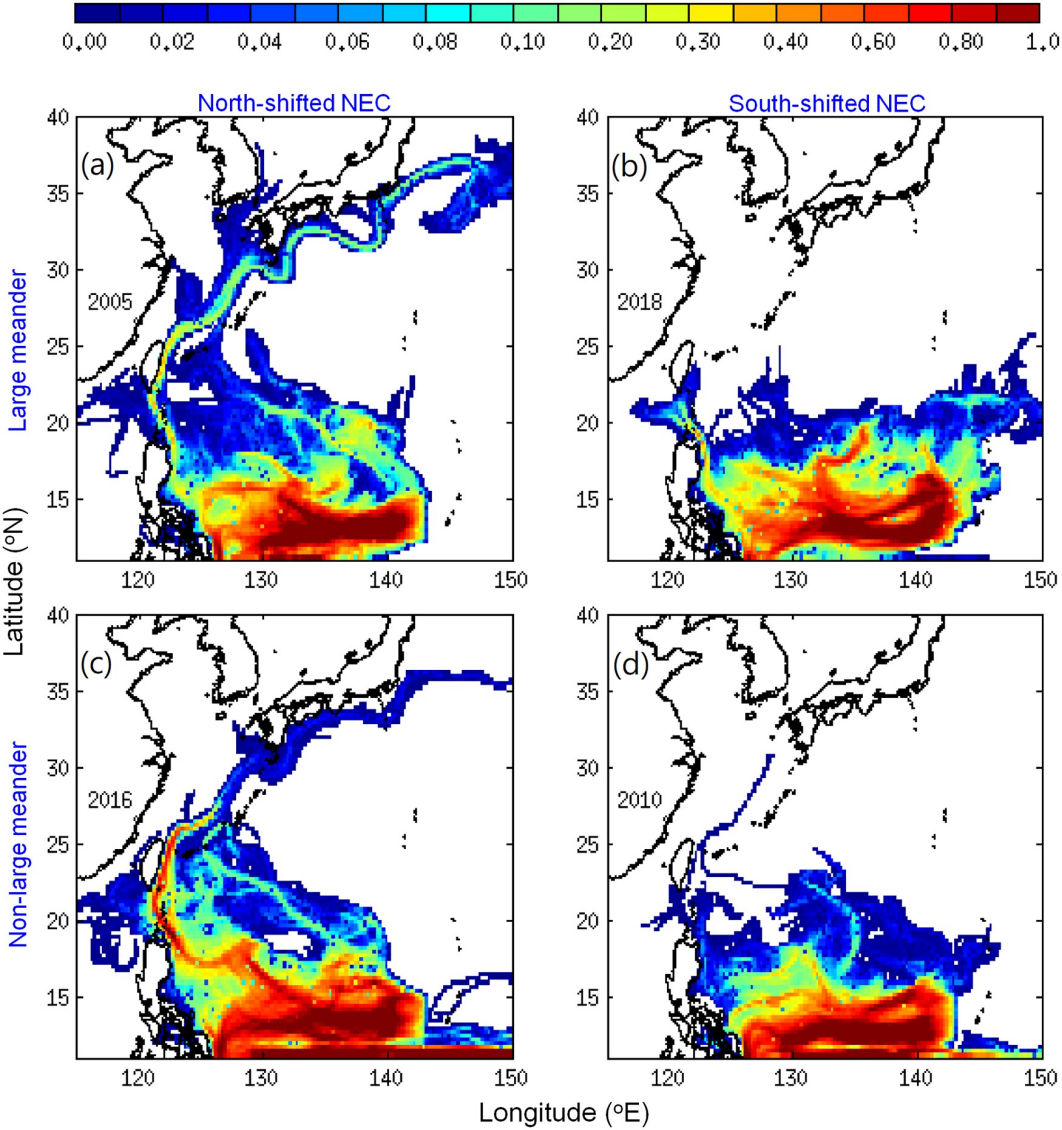
Visitation frequency distributions of v-larvae at the end of January during the early recruitment period in Kuroshio large meander years (a, 2005, b, 2018) and normal years (c, 2016, d, 2010). The unit of visitation frequency was normalized by the total number of released v-larvae.

V-larvae distributions in January during the early recruitment period showed some similarities with the recruitment observations (glass eel catches) (Figs 1 and 3). In the 2018 early period simulation, almost all the v-larvae remained to the south of 25°N and none of the v-larvae arrived at Japan (Fig 3b). In contrast, the v-larvae in the previous Kuroshio large meander event in 2005 could reach the East China Sea, Korea, and the southern coast of Japan. V-larvae also made it to southern Japan in 2016 when there was no large meander. The higher v-larvae arrival to Japan in 2016 compared to 2018 during the early recruitment period was similar to the glass eel recruitment observations (Fig 1c). In 2010 with no large meander, v-larvae also mostly remained to the south of 25°N, with only a few v-larvae entering the Kuroshio in the East China Sea (Fig 3d). More v-larvae were transported into the Kuroshio and reached Japan in 2005 and 2016 when the NEC was shifted north (Fig 3a and 3c), whereas v-larvae were limited to the south with much less arrival to the Kuroshio, East of Taiwan or Japan during the south-positioned NEC conditions in 2010 and 2018 (Fig 3b and 3d). Therefore, v-larvae distribution in the early recruitment period appeared to be primarily influenced by the bifurcation latitude of the NEC, which had also been noticed to affect recruitment success in earlier studies [12, 14].

**Fig 4:**
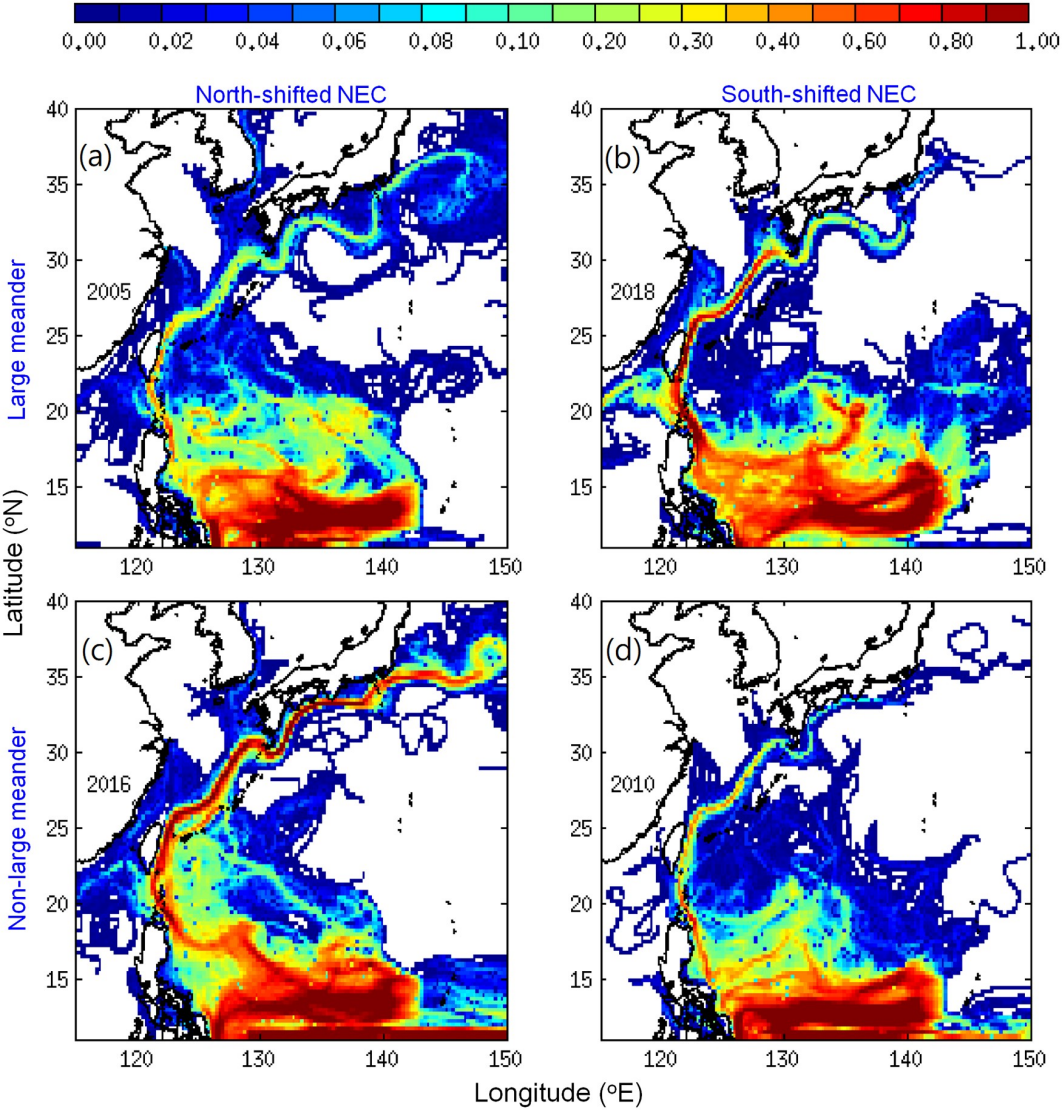
Visitation frequency distributions of v-larvae at the end of April during the late recruitment period in Kuroshio large meander years (a, 2005, b, 2018) and normal years (c, 2016, d, 2010). The unit of visitation frequency was normalized by the total number of released v-larvae.

During April in the late recruitment period, although differences were seen between years, v-larvae could make it to Japan and other recruitment areas in all cases (Fig 4). In 2018, v-larvae arrival to the Kuroshio had increased substantially after January and the amount of v-larvae reaching east of Taiwan and the East China Sea even exceeded 2005, which had more arrival to Japan in the early recruitment period. The significant increase of late-season arrival of v-larvae to Japan in 2018 was similar to the observed glass eel catches that peaked in February to April (Figs 1 and 4); but in this case, the possible effect of some of those glass eels being imported into Japan from other parts of East Asia for use in aquaculture due to low local catches cannot be determined. The Kuroshio large meander caused the v-larvae to be transported offshore in a loop of hundreds of kilometers in 2005 and 2018 (Fig 4a and 4b). While the Kuroshio was in a non-large meander state in 2016 and 2010, v-larvae were present much closer to the coastline of southern Japan (Fig 4c and 4d). Despite the similar general patterns between the simulations and recruitment observations that both showed the late recruitment peaks in 2018 in comparison to 2016, the monthly v-larvae arrival to Japan showed a 1−2 month lag compared to the observations (Fig 1c and 1d). This might have been caused by the settings of the swimming behavior, which are not known to be correct or not due to a lack of observations of swimming by eel larvae in the ocean.

### Role of the Kuroshio large meander

Although the Kuroshio large meander was speculated to be connected to the low eel recruitment in 2018 because of its occurrence when there were lower overall catches of glass eels, the large meander did not necessarily lead to less eel arrival or low recruitment, because a slightly lower overall glass eel catches occurred in 2010 when the Kuroshio took the nearshore path compared to during the previous large meander in 2005 (Fig 1b). Low overall recruitment success to Japan in 2010 (5.0%) was also captured in the simulations, in comparison to the large meander year of 2005 (5.7%) and 2018 (7.6%). The other non-large meander year of 2016, however, showed the highest recruitment success (20.5%) among the four selected years, which also agreed with the glass eel catch observations.

**Fig 5:**
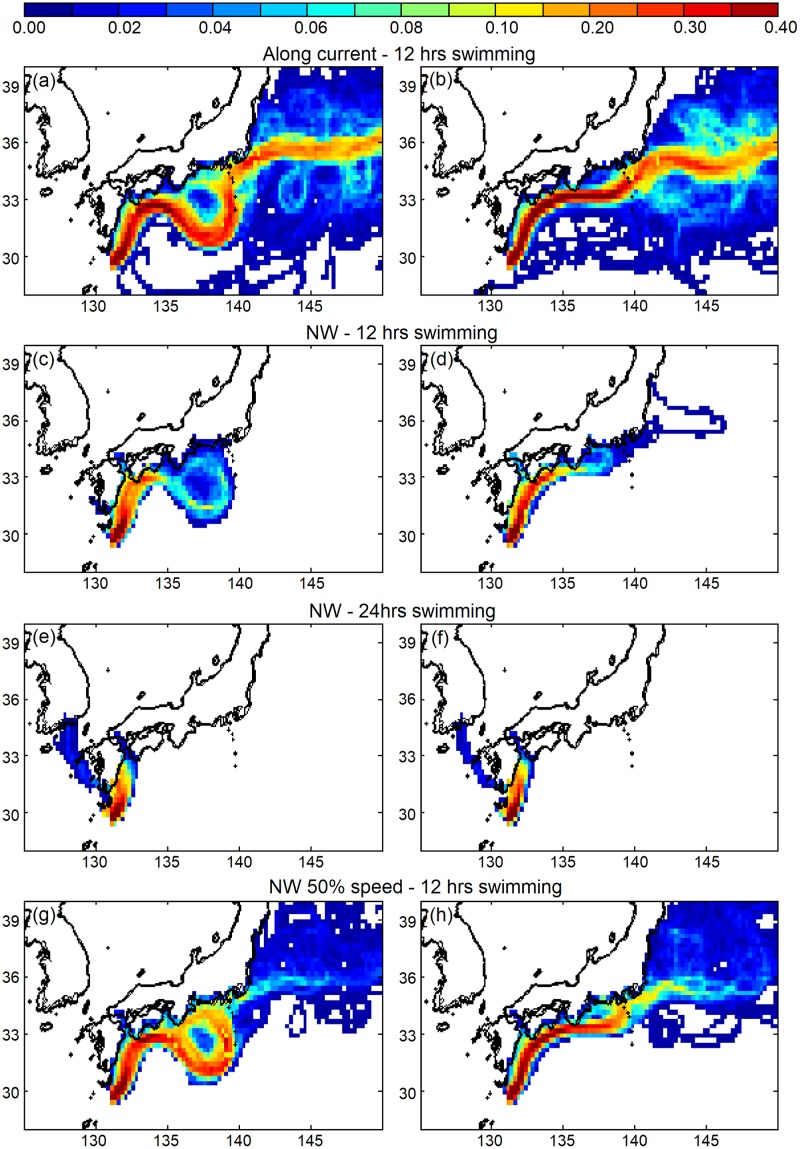
Visitation frequency of v-larvae released within the Kuroshio south of Japan in the large meander years (2005 and 2018, left) and non-large meander years (2010 and 2016, right) with different swimming strategies at the end of the recruitment season: Along current (a, b), northwestward swimming (c, d), 24-hour northwestward swimming (e, f), and half speed northwestward swimming (g, h).

The sensitivity experiments that released v-larvae within the Kuroshio south of Japan examined the effect of the large meander on the larvae crossing out of the Kuroshio (Table 1, Fig 5). V-larvae distribution was sensitive to the Kuroshio large meander as well as the swimming strategies. During the large meander years, many v-larvae followed the path of the Kuroshio meander offshore south of Japan, whereas v-larvae in the non-large meander years tended to take the nearshore path. Even though v-larvae were transported offshore by the Kuroshio large meander, the recirculation formed inside the meander (Fig 2, south of Tokai region) or the effects of swimming could help v-larvae reach nearshore. The number of along-current swimming v-larvae recruiting to the southern Tokai region was higher during the large meander periods (Table 2). If v-larvae performed northwestward directional swimming and had strong swimming effort (long-term swimming and/or fast swimming), they tended to move northwestward and entered the Bungo Channel (between Kyushu and Shikoku) or swam towards Korea (Fig 5c, 5d, 5e and 5f). In that case, the effect of the Kuroshio large meander would be smaller or none, because the v-larvae had quickly detrained from the Kuroshio, and most v-larvae (>80%) would recruit to the Kyushu or Shikoku regions (Table 2). The northwestward swimming, however, led to less recruitment during large meander years in the Tokai region near the meander (see Fig 2), possibly because v-larvae would have to swim against the Kuroshio in the western part of meander region (Table 2). The oriented swimming could enhance the recruitment rate even with the half swimming speed, whereas along current swimming v-larvae were mostly (>90%) transported by the ocean currents without reaching the continent.

**Table 2:** Percentage of successful recruitment to various regions of southern Japan in the swimming sensitivity experiment during the 2 large meander (LM; 2005, 2018) and non-large meander (NLM; 2010, 2016) years. Fail accounted for those v-larvae that did not approach the Japanese shore.

Exp.	Along Current- 12 hrs		NW-12 hours		NW-24 hrs		NW half speed- 12 hrs	
Kuroshio	LM	NLM	LM	NLM	LM	NLM	LM	NLM
Kyushu	0.5	0	47.0	44.4	84.1	90.7	21.4	19.9
Shikoku	1.5	2.5	34.9	35.6	--	0.2	22.0	14.4
Tokai	6.1	2.8	9.3	14.4	--	--	17.7	25.8
Kanto	--	--	--	--	--	--	--	--
Fail	91.9	94.7	8.8	5.6	15.9	9.1	38.9	39.9

### Influence of ocean circulation

**Fig 6:**
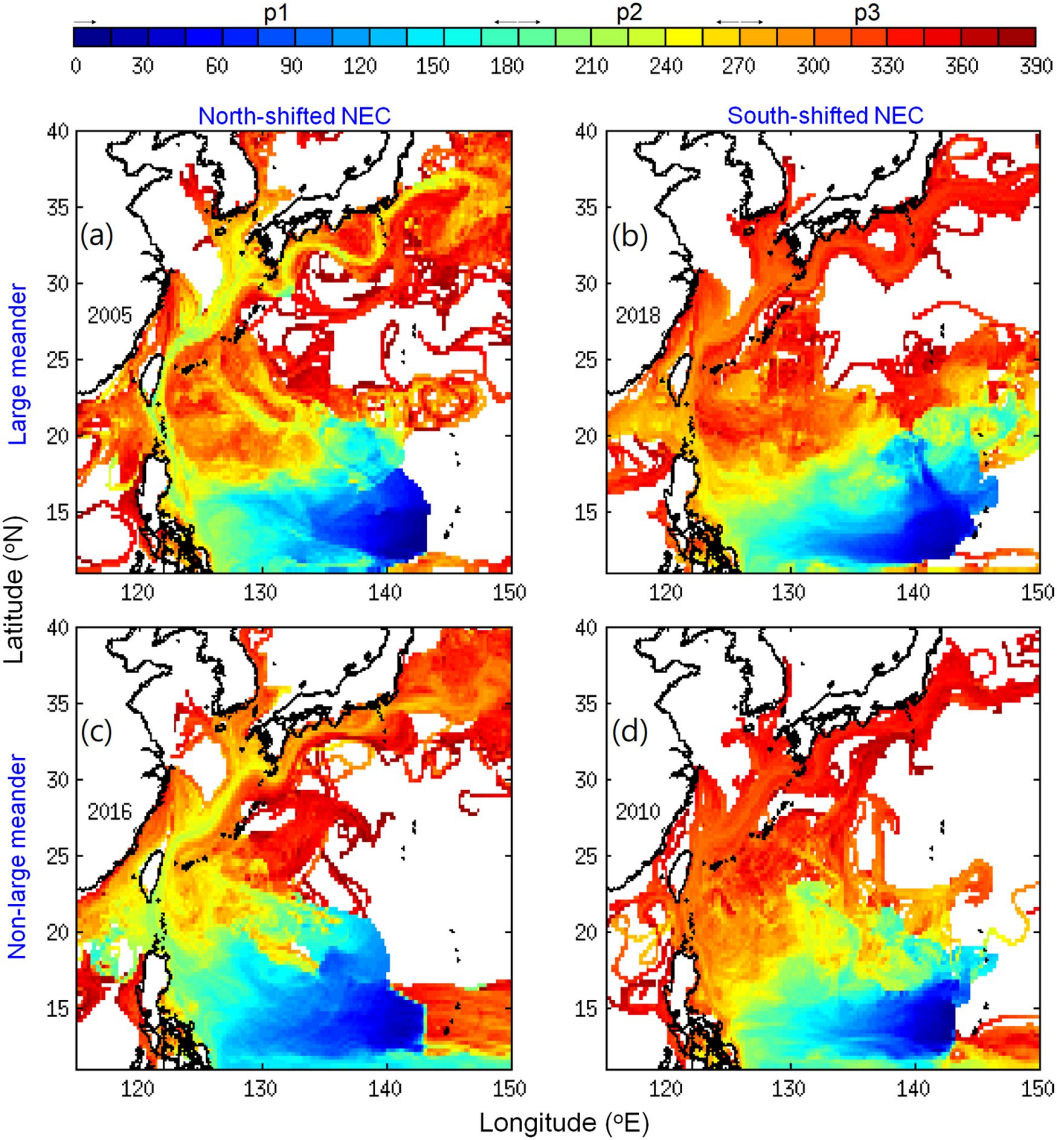
The averaged time taken (days) from the spawning area to each particular grid location within the simulation region. Day 0 to 180 corresponds to the early migration period (p1), day 180 to 270 to the early recruitment period (p2), and after day 270 is the late recruitment period (p3).

**Fig 7:**
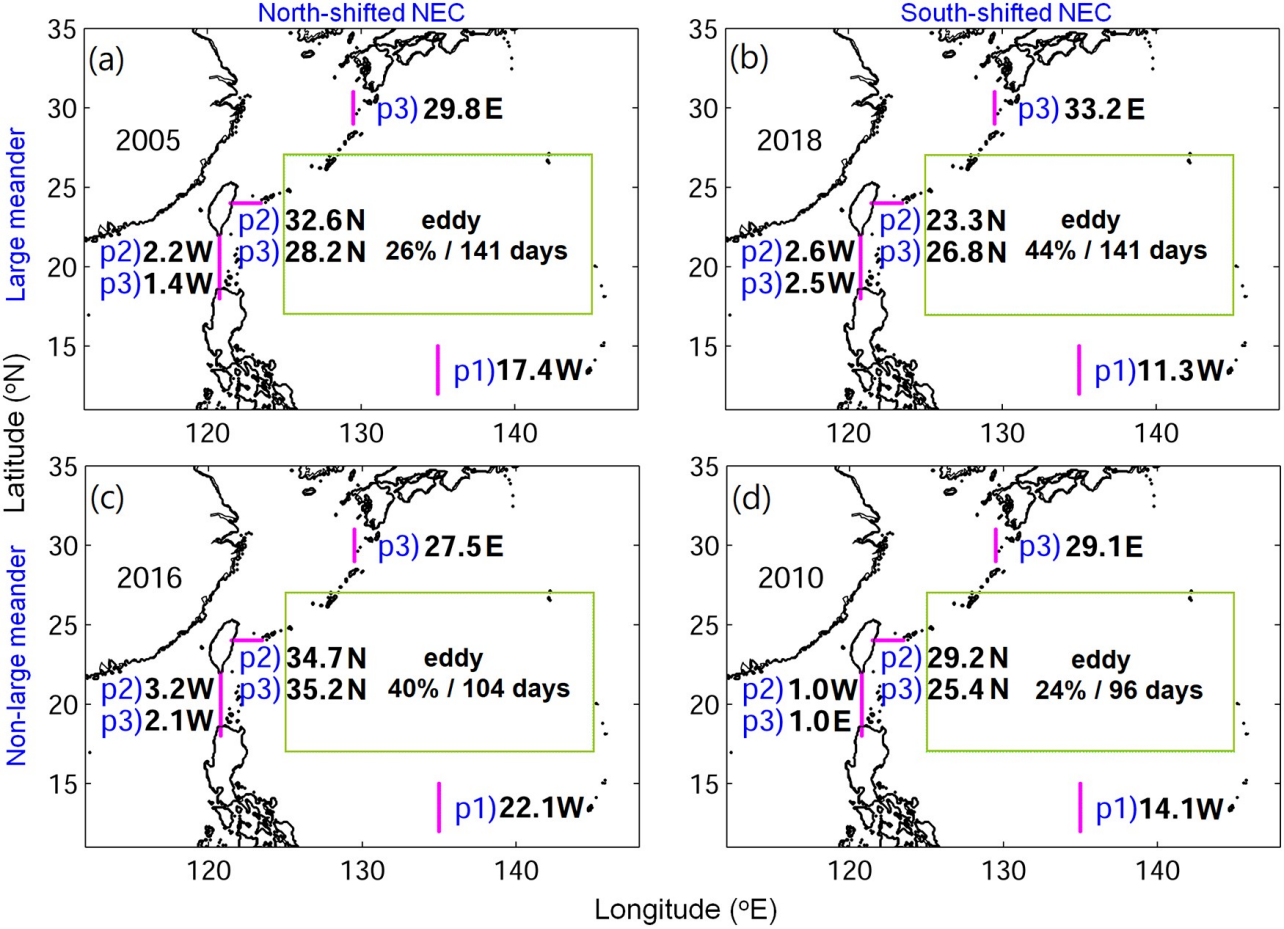
The simulated 50−250 m depth-averaged current speeds (cm/s) and eddy presence (percentage of eddies and average days of retention within the green box) at the corresponding periods of v-larvae passing through. Period 1 (p1) is early migration from May to October, period 2 (p2) is early recruitment from November to coming January, and period 3 (p3) is late recruitment from February to April. Magenta lines indicate the sections used for calculating mean velocity. N, W, and E next the numbers indicate the current flow directions of northward, westward, and eastward, respectively.

As the low eel catch also occurred at the upstream location of Taiwan, the cause of an extremely low catch in the early recruitment period and the sudden increase and late arrival in the late recruitment period likely originated in the offshore ocean upstream of the Kuroshio region (Fig 2). Considering that the estimated eel larvae migration to East Asia generally takes 6 months or longer [12, 45], and the ocean conditions were highly time-dependent, the average time taken from the spawning area to move past each particular region was calculated (Fig 6). We next investigated the ocean current conditions that v-larvae encountered during each part of their migration (Fig 7). After departing from the spawning area, v-larvae were transported by the NEC during the first 180 days of the early migration period (p1). They then arrived along the eastern Philippines and Luzon Strait at the early (p2) and late (p3) recruitment seasons. V-larvae were found in the STCC region during the entire simulation (p1, p2, p3), they entered the Kuroshio and arrived to the east of Taiwan in the early and late recruitment seasons (p2, p3), and reached south of Japan around the late recruitment season (p3).

The regional ocean current velocities were calculated according to the different periods/locations during the migration (Figs 6 and 7). The NEC near the spawning area (12−15°N) was generally weaker during the south-shifted periods (2018 and 2010 in Fig 7b and 7d) because the fast-flowing core of the NEC was shifted to the south of the spawning area. The NEC was particularly weak in 2018 (11.3 cm/s) and was only about 50% of the speed of 2016 (22.1 cm/sec) (Fig 7b and 7c). It was also slow in 2010 (14 cm/s) and moderately faster in 2005 (17.4 cm/sec). The slow-moving NEC, therefore, could not transport v-larvae efficiently to enable normal recruitment timing to occur. The effect of this was clear in Fig 3 that shows larvae reaching Japan in the faster NEC years (2005 and 2016) during the early recruitment period, but not in the slow NEC years (2010 and 2018).

**Fig 8:**
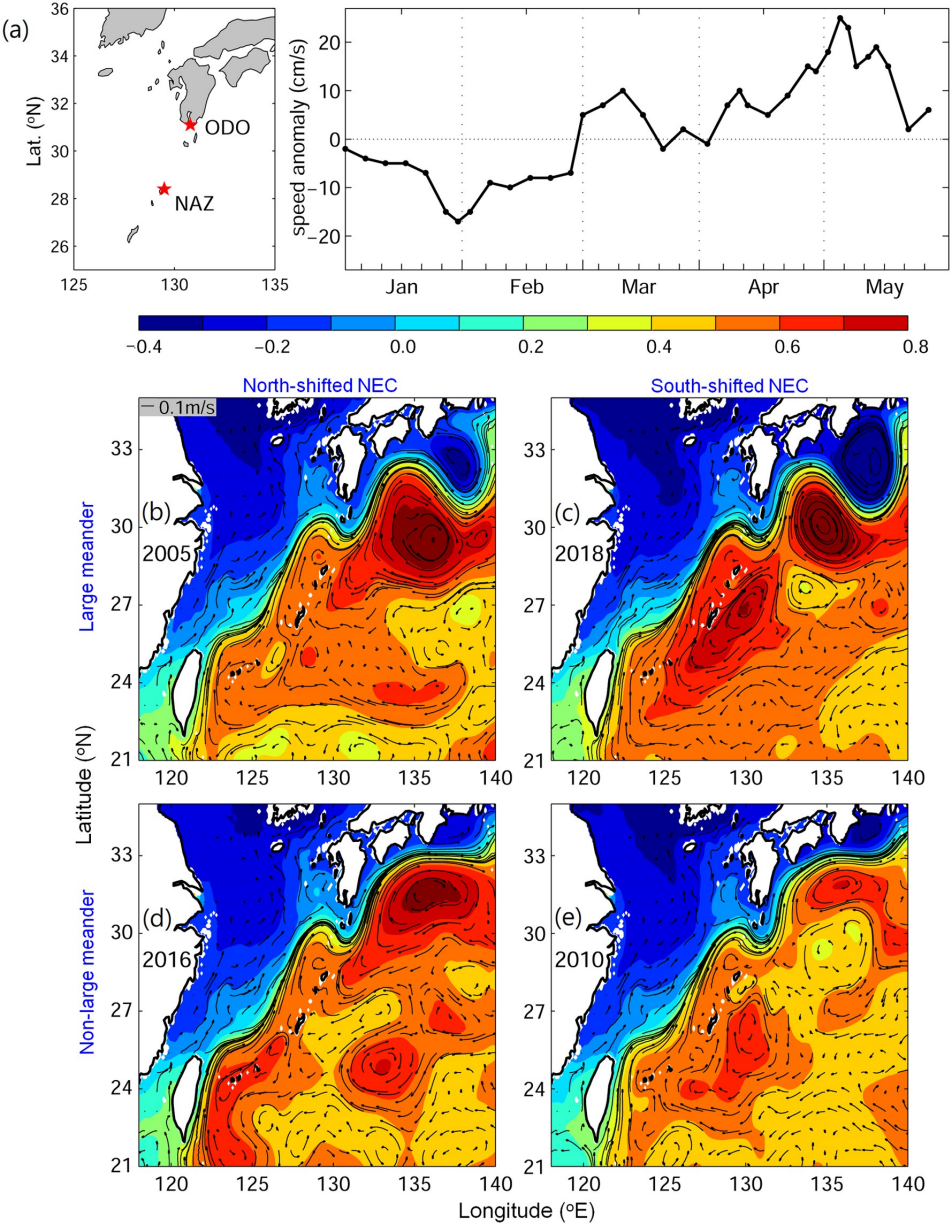
The 2018 Kuroshio geostrophic velocity anomaly (cm/s) south of Japan derived from tide gauge stations Oodori (ODO) and Naze (NAZ) (a). Plots of the sea surface height (b−e, color shading shows height in meters) and 50−250 m averaged ocean currents (vectors, m/s) in the late recruitment season (period 3, February to April). Red shading and clockwise circulation often indicate the presence of warm eddies, and blue and anti-clockwise circulation corresponds to cold eddies.

Part of the v-larvae entered the STCC eddy region (Figs 3, 4 and 6) and could become trapped and transported by eddies [31]. The percentage of v-larvae that entered the STCC eddy zone was highest in 2018 (44%) followed by 2016 (40%), 2005 (26%) and 2010 (24%). The average retention time in eddies was about 141 days for both 2018 and 2005. A shorter retention time was observed in 2016 (104 days) and 2010 (96 days). Hence, more eddies and longer retention time in 2018 may have resulted in later arrival to the Kuroshio. While v-larvae entrained into the northward Kuroshio east of Philippines, some soon became transported into the Luzon Strait and into to the South China Sea without continuing northward. The Luzon Strait intrusion in 2018 was stronger than in 2005 and 2010, and was comparable to during 2016, so part of the v-larvae were transported into the South China Sea (Figs 3, 4 and 7). The Kuroshio in the early 2018 recruitment period (p2) was 20−30% weaker than the other selected years, which was unfavorable for transporting v-larvae downstream. As a result, the ocean conditions in the early migration period p1 and early recruitment period p2 were unfavorable for transporting v-larvae, leading to the slower downstream transport to East Asia in the early recruitment season of 2018 (Fig 3). In the late recruitment period (p3) of 2018 when many v-larvae had entrained into the Kuroshio (Fig 6), the Kuroshio was strengthened with higher speed especially to the south of Japan (Fig 7). The strengthening of the Kuroshio south of Japan was also recorded in the time-series of tide gauge observations (Fig 8a), which showed the Kuroshio surface speed increased in p3 from the beginning of February to early May by about 40 cm/s. The fast-flowing Kuroshio could, therefore, transport v-larvae downstream efficiently, resulting in the increased arrival in late recruitment season (Fig 4).

## Discussion

**Fig 9:**
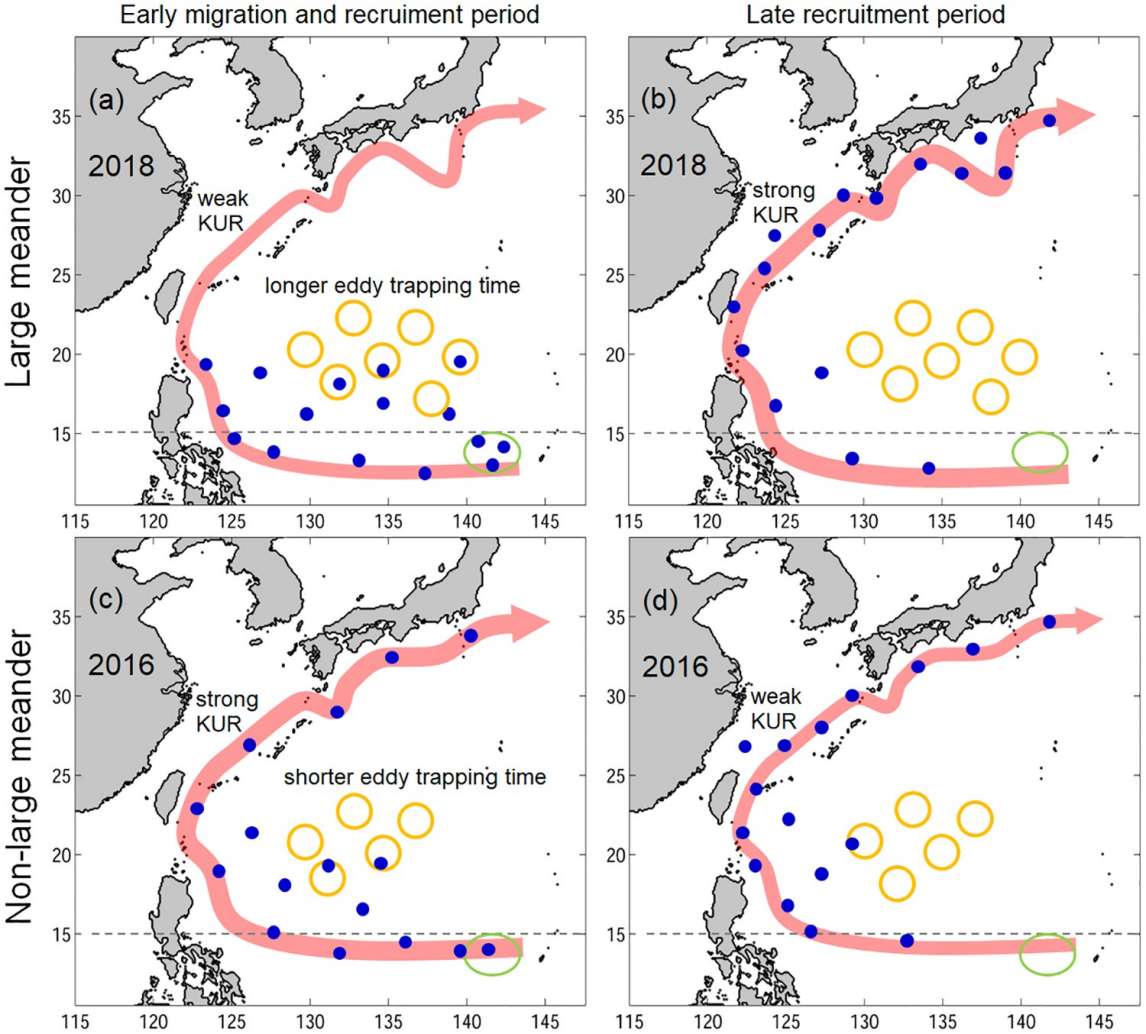
Schematic diagram illustrating the ocean conditions and v-larvae distributions for the 2018 large meander (a,b) and 2016 non-large meander (c,d) years during the early migration and recruitment season (a, c), and late recruitment period (b, d). Yellow circles represent eddies, blue dots represent v-larvae, the width of the red shading arrows reflects the strength of the Kuroshio (KUR), and green circle marks the spawning area in the North Equatorial Current (NEC). The dotted line enables comparison of the relative position of the NEC.

The present study explored the potential impact of the Kuroshio large meander and ocean circulation on the unusual pattern of eel recruitment in 2018 using a three-dimensional particle-tracking method (with horizontal swimming and vertical DVM). The extremely low glass eel catches observed in the early recruitment season in Japan and the higher catches in the late recruitment period in 2018 were captured by the simulations, suggesting that ocean circulation plays a crucial role in the timing of arrival of glass eels to Japan. No direct linkage between the large meander and lower recruitment was found in the simulations, because v-larvae could be transported downstream efficiently and reached southern Japan in the early recruitment period when the NEC was north-shifted in 2005 (large meander) and 2016 (no large meander), whereas v-larvae showed little or no arrival to Japan when the NEC was south-shifted in 2010 (no large meander) and 2018 (large meander). In 2018, the lower eel catches in the early recruitment season occurred when there was a weak NEC, more STCC eddies and longer retention in eddies, strong Luzon Strait intrusion, and a weak Kuroshio, which suggests there were unfavorable conditions for transporting v-larvae towards East Asia. The Kuroshio was strengthened in the late recruitment season, which transported v-larvae downstream more efficiently, resulting in increased eel catches in the late recruitment period. A schematic diagram (Fig 9) summarizes the apparent effect of ocean circulation on the 2018 late recruitment occurrence in comparison to in 2016 when there was no large meander.

The Kuroshio large meander happened to occur when the unusual eel recruitment was observed in 2018. Although our experiments suggested that the Kuroshio large meander did not necessarily lead to less recruitment or late arrival, they did suggest that NEC bifurcation played a more important role in affecting the recruitment. The change of ocean conditions from the early to the late recruitment period was likely caused by a phase change of the Philippines–Taiwan Oscillation (PTO), which is based on the difference between the tropical and subtropical wind stress curl [46]. PTO was changing from a negative phase to a positive phase during the 2018 recruitment season, and the major ocean features corresponded to the typical characteristics of the PTO, such as the weak NEC and Kuroshio in the early recruitment season while PTO was in a negative phase and the strengthening of the Kuroshio in the late recruitment period when PTO turned positive. The ocean circulation in the positive PTO phase is more favorable for Japanese eel larval migration due to the northward shifted and faster NEC near the spawning area and the stronger Kuroshio, while the negative phase with the opposite oceanic conditions is less favorable for recruitment [12, 14]. Therefore, the low glass eel catches in the early recruitment period and the increased catches in the late recruitment period in 2018 could have been caused by the phase change of the PTO. However, the relationship between the phase change of PTO and late recruitment should be explored further, because the observed monthly glass eel catch data were only available after 2016, and there was only one PTO phase change event that occurred in our study (2018).

Separate from the PTO, more warm eddies to the northeast of the Okinawa Islands might also contribute to the strengthening of the Kuroshio in the East China Sea and south of Japan [47]. During the 2018 late recruitment period, more warm eddies were accumulated and merged into a big warm eddy on the east side of the Ryukyu Islands (126−132°E, 25−29°N, Fig 8c). In contrast, fewer warm eddies in 2016 occurred when there was a weak Kuroshio (Figs 7 and 8).

A previous study also documented the late arrival of glass eels to the Sagami River of Japan (139.4°E, 35.3°N) in 2010 [48] when the NEC was shifted to the south, but there was no Kuroshio large meander. The Kuroshio meander will transport eel larvae hundreds of kilometers offshore if they remain in the current, making it more difficult to reach the coast in some areas. Although a recruitment reduction was observed in 2018 as well as during the previous large meander in 2005, enhanced recruitment in the Tokai region was also recorded in an earlier large meander event in the 1990s [49], and it was proposed that eel larvae could be transported by the recirculation inside the meander loop (Fig 2). The sensitivity experiments that released v-larvae south of Japan for examining the effect of large meander also showed that along current swimming v-larvae could be transported by the recirculation and led to more recruitment near the Tokai region (Fig 5, Table 2). This suggested that oriented swimming, which was carefully evaluated in a previous study [24], could enhance the recruitment success to Japan, and that recruitment to the Tokai region could be reduced during large meander periods if northwest swimming was used. So it is difficult to conclude that the Kuroshio large meander has a positive or negative effect on eel or other fisheries species recruitment without further observations and detailed analyses, especially if the larvae must actively swim to get out of the Kuroshio to reach coastal waters. Various studies have shown that larval swimming by anguillid eels seems to be required to cross out of the western boundary currents such as the Kuroshio [24] and the Gulf Stream [50]. Although it is not yet known if or how the larvae use directional swimming, but eels including the Japanese eel were found to be able to sense magnetic fields [51–53], which may serve as a compass for directional swimming.

Apart from the Japanese eel, the Kuroshio large meander was also reported to influence other fisheries species, such as whitebait (mostly engraulid fish larvae) and skipjack tuna (information from Japan Fisheries Agency based on the previous large meander event in 2005: http://nrifs.fra.affrc.go.jp/ResearchCenter/3_FOME/kuroshio/index.html). Instead of overall enhancement or reduction, the recruitment of both species seemed to be redistributed. Whitebait and skipjack were reported to have recruitment reductions in the Tokai region, but increases in areas further downstream along the Kuroshio. Late arrival of whitebait was also observed in the eastern Shikoku region. These are species that occur relatively close to Japan, and they do not make long larval migrations from far offshore like the Japanese eel does.

What the larval transport modelling studies such as the present one have shown is that interannual ocean circulation changes may have an influence on the patterns or timing of glass eel recruitment. One aspect is that there may have been a long-term change in ocean circulation, such as a weakening and southward shifting of the NEC and a weakening of the Kuroshio that may reduce glass eel recruitment to East Asia [13]. Other aspects are that the latitude of spawning seems to be related to the location of the salinity front caused by tropical rainfall across the NEC [54]. Shifts in spawning latitudes have been tested in simulations along with comparisons of El Niño and other years [15, 55], which can affect levels of successful transport of larvae to East Asia. However, as in the present study, the studies on the latitude of NEC bifurcation [12], or as reflected by the PTO [12, 14], suggest that bifurcation latitude might have an important influence on recruitment, unless active swimming might be able to mitigate against some of that, which had been proposed in other species [56, 57]. In addition, biological factors that were not considered in the present study, such as increase in mortality during early recruitment, environmental changes within the spawning area affecting early larval survival [17], or later spawning etc, are also factors that might have the potential to cause late recruitment.

While this first study on the effect of the Kuroshio large meander did not find evidence of this periodically occurring ocean circulation feature affecting the recruitment using a transport modelling simulation approach, this subject should be investigated further. This study did however, find indications that other ocean circulation features have the potential to influence recruitment, which is consistent with the previous studies mentioned above. Therefore, further studies are needed to evaluate the linkages between actual recruitment fluctuations based on glass eel abundances and recruitment timing, ocean conditions, and simulations of these conditions to better understand the factors affecting the recruitment of the Japanese eel.

## References

[1] Aida K, Tsukamoto K, Yamauchi K. Eel Biology. Springer Japan 2003:497.

[2] Tesch F. The eel. Blackwell publishing 2003:408.

[3] Tsukamoto K. Oceanic migration and spawning of anguillid eels. Journal of fish biology. 2009;74(9):1833–52. 10.1111/j.1095-8649.2009.02242.x . 20735675

[4] Kuroki M, Miller MJ, Tsukamoto K. Diversity of early life history traits in freshwater eels and the evolution of their oceanic migrations. Can J Zool. 2014;92:749–70. 10.1139/cjz-2013-0303

[5] Miller MJ, Tsukamoto K. The ecology of oceanic dispersal and survival of anguillid leptocephali. Canadian Journal of Fisheries and Aquatic Sciences. 2017;74(6):958–71. 10.1139/cjfas-2016-0281

[6] ICES. Reports of the Eifac/ICES working group on eels (WGEEL), 18–22 March 2013 in Sukarietta, Spain, 4–10 September 2013 in Copenhagen, Denmark ICES CM 2013/ACOM:18 253 pp. 2013.

[7] Dekker W. Did lack of spawners cause the collapse of the European eel, Anguilla anguilla? Fisheries Management and Ecology. 2003;10(6):365–76. 10.1111/j.1365-2400.2003.00352.x

[8] Baltazar-Soares M, Biastoch A, Harrod C, Hanel R, Marohn L, Prigge E, et al Recruitment collapse and population structure of the European eel shaped by local ocean current dynamics. Current biology: CB. 2014;24(1):104–8. 10.1016/j.cub.2013.11.031 . 24374306

[9] Bonhommeau S, Chassot E, Planque B, Rivot E, Knap A, Le Pape O. Impact of climate on eel populations of the Northern Hemisphere. Marine Ecology Progress Series. 2008;373:71–80. 10.3354/meps07696

[10] Friedland KD, Miller MJ, Knights B. Oceanic changes in the Sargasso Sea and declines in recruitment of the European eel. ICES Journal of Marine Science. 2007;64(3):519–30. 10.1093/icesjms/fsm022

[11] Knights B. A review of the possible impacts of long-term oceanic and climate changes and fishing mortality on recruitment of anguillid eels of the Northern Hemisphere. Science of The Total Environment. 2003;310(1–3):237–44. 10.1016/S0048-9697(02)00644-7 12812748

[12] Chang Y-L, Sheng J, Ohashi K, Béguer-Pon M, Miyazawa Y. Impacts of Interannual Ocean Circulation Variability on Japanese Eel Larval Migration in the Western North Pacific Ocean. PLoS ONE. 2015;10(12):e0144423 10.1371/journal.pone.0144423 26642318 PMC4671650

[13] Chang Y-LK, Miyazawa Y, Miller MJ, Tsukamoto K. Potential impact of ocean circulation on the declining Japanese eel catches. Scientific Reports. 2018;8(1):5496 10.1038/s41598-018-23820-6 29615739 PMC5883023

[14] Hsu AC, Xue H, Chai F, Xiu P, Han Y-S. Variability of the Pacific North Equatorial Current and its implications on Japanese eel (Anguilla japonica) larval migration. Fisheries Oceanography. 2017;26(3):251–67. 10.1111/fog.12189

[15] Kim H, Kimura S, Shinoda A, Kitagawa T, Sasai Y, Sasaki H. Effect of El Niño on migration and larval transport of the Japanese eel (Anguilla japonica). ICES Journal of Marine Science: Journal du Conseil. 2007;64(7):1387–95. 10.1093/icesjms/fsm091

[16] Zenimoto K, Sasai Y, Sasaki H, Kimura S. Estimation of larval duration in Anguilla spp., based on cohort analysis, otolith microstructure, and Lagrangian simulations. Marine Ecology Progress Series. 2011;438:219–28. 10.3354/meps09255

[17] Miller MJ, Feunteun E, Tsukamoto K. Did a “perfect storm” of oceanic changes and continental anthropogenic impacts cause northern hemisphere anguillid recruitment reductions? ICES Journal of Marine Science. 2016;73(1):43–56. 10.1093/icesjms/fsv063

[18] Jacoby D, Gollock, M. Anguilla japonica. The IUCN Red List of Threatened Species Version 2015–3. 2014.

[19] Dekker W. Slipping through our hands: population dynamics of the European eel. IJmuiden: s.n.; 2004.

[20] Tsukamoto K. Oceanic biology: Spawning of eels near a seamount. Nature. 2006;439(7079):929-. http://www.nature.com/nature/journal/v439/n7079/suppinfo/439929a_S1.html 16495988 10.1038/439929a

[21] Tsukamoto K, Chow S, Otake T, Kurogi H, Mochioka N, Miller MJ, et al Oceanic spawning ecology of freshwater eels in the western North Pacific. Nature communications. 2011;2:179 10.1038/ncomms1174 21285957 PMC3105336

[22] Qiu B, Chen S. Multidecadal Sea Level and Gyre Circulation Variability in the Northwestern Tropical Pacific Ocean. Journal of Physical Oceanography. 2012;42(1):193–206. 10.1175/jpo-d-11-061.1

[23] Qiu B, Rudnick DL, Cerovecki I, Cornuelle BD, Chen S, Schönau MC, et al The Pacific North Equatorial Current: New insights from the origins of the Kuroshio and Mindanao Currents (OKMC) Project. Oceanography. 2015;28(4):24–33. 10.5670/oceanog.2015.78.

[24] Chang Y-LK, Miller MJ, Tsukamoto K, Miyazawa Y. Effect of larval swimming in the western North Pacific subtropical gyre on the recruitment success of the Japanese eel. PLOS ONE. 2018;13(12):e0208704 10.1371/journal.pone.0208704 30571715 PMC6301772

[25] Kawabe M. Variations of Current Path, Velocity, and Volume Transport of the Kuroshio in Relation with the Large Meander. Journal of Physical Oceanography. 1995;25(12):3103–17. 10.1175/1520-0485(1995)025<3103:VOCPVA>2.0.CO;2

[26] Kawabe M. Variations of the Kuroshio in the Southern Region of Japan: Conditions for Large Meander of the Kuroshio. J Oceanogr. 2005;61(3):529–37. 10.1007/s10872-005-0060-0

[27] Usui N, Tsujino H, Fujii Y, Kamachi M. Generation of a trigger meander for the 2004 Kuroshio large meander. Journal of Geophysical Research: Oceans. 2008;113(C1). 10.1029/2007JC004266

[28] Usui N, Tsujino H, Nakano H, Matsumoto S. Long-term variability of the Kuroshio path south of Japan. J Oceanogr. 2013;69(6):647–70. 10.1007/s10872-013-0197-1

[29] Endoh T, Tsujino H, Hibiya T. The Effect of Koshu Seamount on the Formation of the Kuroshio Large Meander South of Japan. Journal of Physical Oceanography. 2011;41(9):1624–9. 10.1175/jpo-d-11-074.1

[30] Zenimoto K, Kitagawa T, Miyazaki S, Sasai Y, Sasaki H, Kimura S. The effects of seasonal and interannual variability of oceanic structure in the western Pacific North Equatorial Current on larval transport of the Japanese eel Anguilla japonica. Journal of fish biology. 2009;74(9):1878–90. 10.1111/j.1095-8649.2009.02295.x 20735678

[31] Chang Y-L, Miyazawa Y, Béguer-Pon M. The dynamical impact of mesoscale eddies on migration of Japanese eel larvae. PLOS ONE. 2017;12(3):e0172501 10.1371/journal.pone.0172501 28253293 PMC5333816

[32] Chang Y-LK, Miyazawa Y, Béguer-Pon M, Han Y-S, Ohashi K, Sheng J. Physical and biological roles of mesoscale eddies in Japanese eel larvae dispersal in the western North Pacific Ocean. Scientific Reports. 2018;8(1):5013 10.1038/s41598-018-23392-5 29567996 PMC5864879

[33] Marshall J, Plumb A. Atmosphere, Ocean and Climate Dynamics. Academic Press 2007:344.

[34] Miyazawa Y, Zhang R, Guo X, Tamura H, Ambe D, Lee J-S, et al Water mass variability in the western North Pacific detected in a 15-year eddy resolving ocean reanalysis. J Oceanogr. 2009;65(6):737–56. 10.1007/s10872-009-0063-3

[35] Ohashi K, Sheng J. Investigating the Effect of Oceanographic Conditions and Swimming Behaviours on the Movement of Particles in the Gulf of St. Lawrence Using an Individual-Based Numerical Model. Atmosphere-Ocean. 2015:1–21. 10.1080/07055900.2015.1090390

[36] Press W, Teukolsky S, Vetterling W, Flannery B. Numerical recipes in Fortran 77: the art of scientific computing. Cambridge University Press 1992.

[37] Béguer-Pon M, Shan S, Thompson KR, Castonguay M, Sheng J, Dodson JJ. Exploring the role of the physical marine environment in silver eel migrations using a biophysical particle tracking model. ICES Journal of Marine Science: Journal du Conseil. 2015 10.1093/icesjms/fsv169

[38] Béguer-Pon M, Ohashi K, Sheng J, Castonguay M, Dodson JJ. Modeling the migration of the American eel in the Gulf of St. Lawrence. Marine Ecology Progress Series. 2016;549:183–98. 10.3354/meps11706

[39] Aoyama J, Watanabe S, Miller MJ, Mochioka N, Otake T, Yoshinaga T, et al Spawning Sites of the Japanese Eel in Relation to Oceanographic Structure and the West Mariana Ridge. PLoS ONE. 2014;9(2):e88759 10.1371/journal.pone.0088759 24551155 PMC3923831

[40] Tsukamoto K. Discovery of the spawning area for Japanese eel. Nature. 1992;356(6372):789–91.

[41] Tsukamoto K, Umezawa A. Early life history and oceanic migration of the eel, Anguilla japonica. La Mer (Bull Soc franco-jap OceÂanogr, Tokyo). 1990;28:188–98.

[42] Tosi L, Spampanato A, Sola C, Tongiorgi P. Relation of water odour, salinity and temperature to ascent of glass-eels, Anguilla anguilla (L.): a laboratory study. Journal of fish biology. 1990;36(3):327–40. 10.1111/j.1095-8649.1990.tb05613.x

[43] Okubo A. Horizontal dispersion of floatable particles in the vicinity of velocity singularities such as convergences. Deep Sea Research and Oceanographic Abstracts. 1970;17(3):445–54. 10.1016/0011-7471(70)90059-8.

[44] Weiss J. The dynamics of enstrophy transfer in two-dimensional hydrodynamics. Physica D: Nonlinear Phenomena. 1991;48(2):273–94. 10.1016/0167-2789(91)90088-Q.

[45] Shinoda A, Aoyama J, Miller M, Otake T, Mochioka N, Watanabe S, et al Evaluation of the larval distribution and migration of the Japanese eel in the western North Pacific. Rev Fish Biol Fisheries. 2011;21(3):591–611. 10.1007/s11160-010-9195-1

[46] Chang Y-L, Oey L-Y. The Philippines–Taiwan Oscillation: Monsoonlike Interannual Oscillation of the Subtropical–Tropical Western North Pacific Wind System and Its Impact on the Ocean. Journal of Climate. 2012;25(5):1597–618. 10.1175/JCLI-D-11-00158.1

[47] Soeyanto E, Guo X, Ono J, Miyazawa Y. Interannual variations of Kuroshio transport in the East China Sea and its relation to the Pacific Decadal Oscillation and mesoscale eddies. Journal of Geophysical Research: Oceans. 2014;119(6):3595–616. 10.1002/2013JC009529

[48] Aoyama J, Shinoda A, Yoshinaga T, Tsukamoto K. Late arrival of Anguilla japonica glass eels at the Sagami River estuary in two recent consecutive year classes: ecology and socio-economic impacts. Fisheries Science. 2012;78(6):1195–204. 10.1007/s12562-012-0544-y

[49] Iinuma N. The Japanese eel catch in 2005. Shizuoka prefecture fisheries experiment station Hamanako branch Report. 2005:8.

[50] Rypina II, Llopiz JK, Pratt LJ, Susan Lozier M. Dispersal pathways of American eel larvae from the Sargasso Sea. Limnology and Oceanography. 2014;59(5):1704–14. 10.4319/lo.2014.59.5.1704

[51] Nishi T, Kawamura G, Matsumoto K. Magnetic sense in the Japanese eel, Anguilla japonica, as determined by conditioning and electrocardiography. The Journal of experimental biology. 2004;207(Pt 17):2965–70. 10.1242/jeb.01131 . 15277551

[52] Durif CMF, Browman HI, Phillips JB, Skiftesvik AB, Vøllestad LA, Stockhausen HH. Magnetic Compass Orientation in the European Eel. PLoS ONE. 2013;8(3):e59212 10.1371/journal.pone.0059212 23554997 PMC3598651

[53] Cresci A, Paris CB, Durif CMF, Shema S, Bjelland RM, Skiftesvik AB, et al Glass eels (Anguilla anguilla) have a magnetic compass linked to the tidal cycle. Science Advances. 2017;3(6). 10.1126/sciadv.1602007 28630895 PMC5466372

[54] Kimura S, Tsukamoto K. The salinity front in the North Equatorial Current: A landmark for the spawning migration of the Japanese eel (Anguilla japonica) related to the stock recruitment. Deep Sea Research Part II: Topical Studies in Oceanography. 2006;53(3):315–25. 10.1016/j.dsr2.2006.01.009.

[55] Miller M, Kimura S, Friedland K, Knight B, Kim H, Jellyman D, et al Review of ocean-atmospheric factors in the Atlantic and Pacific oceans influencing spawning and recruitment of anguillid eels. American Fisheries Society Symposium 2009;69:231–49.

[56] Scott R, Marsh R, Hays GC. A little movement orientated to the geomagnetic field makes a big difference in strong flows. Marine Biology. 2012;159(3):481–8. 10.1007/s00227-011-1825-1

[57] Putman NF, Verley P, Shay TJ, Lohmann KJ. Simulating transoceanic migrations of young loggerhead sea turtles: merging magnetic navigation behavior with an ocean circulation model. The Journal of experimental biology. 2012;215(11):1863 10.1242/jeb.067587 22573765

